# Correction: Rapid synthesis of internal peptidyl α-ketoamides by on resin oxidation for the construction of rhomboid protease inhibitors

**DOI:** 10.1039/d1ra90086b

**Published:** 2021-03-01

**Authors:** Tim Van Kersavond, Raphael Konopatzki, Merel A. T. van der Plassche, Jian Yang, Steven H. L. Verhelst

**Affiliations:** Leibniz Institute for Analytical Sciences ISAS, e.V. Otto-Hahn-Str. 6b 44227 Dortmund Germany; KU Leuven, Department of Cellular and Molecular Medicine, Laboratory of Chemical Biology Herestr. 49 box 802 3000 Leuven Belgium steven.verhelst@kuleuven.be

## Abstract

Correction for ‘Rapid synthesis of internal peptidyl α-ketoamides by on resin oxidation for the construction of rhomboid protease inhibitors’ by Tim Van Kersavond *et al.*, *RSC Adv.*, 2021, **11**, 4196–4199, DOI: 10.1039/D0RA10614C.

The authors regret that an incorrect version of Fig. 1 was presented in the original manuscript. The R-group in compound 7 was incorrectly indicated as (CH_2_)_5_Ph. The corrected version of the figure with the R group as (CH_2_)_4_Ph is shown below.

**Fig. 1:**
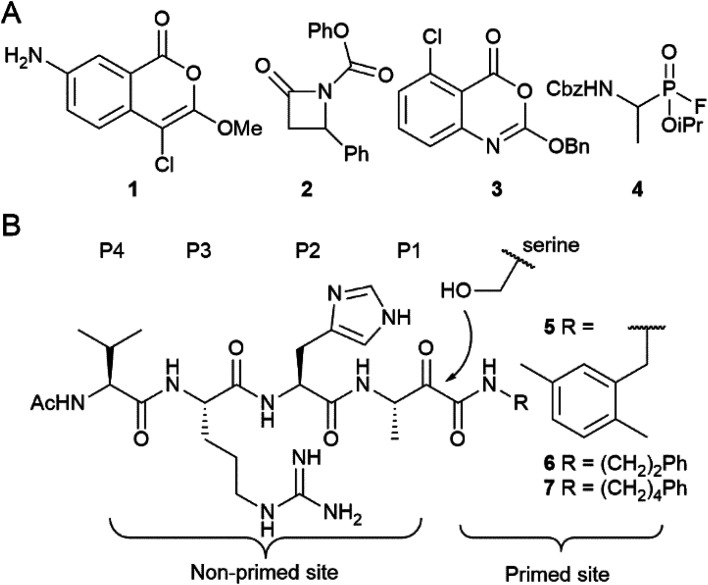
Examples of rhomboid inhibitors. (A) 4-Chloro-isocoumarins (1), β-lactams (2), benzoxazinones (3) and fluorophosphonates (4). (B) α-Ketoamide rhomboid inhibitors (5–7). The peptidic element in the non-primed site is indicated with the P1–P4 position according to the Schechter and Berger protease substrate nomenclature.^1^

The Royal Society of Chemistry apologises for these errors and any consequent inconvenience to authors and readers.

## Supplementary Material
